# Interplay of plasma Oxytocin and oxytocin receptor gene methylation levels on empathy in older adults

**DOI:** 10.1038/s41598-025-07353-3

**Published:** 2025-07-18

**Authors:** Rebecca J. Polk, Kylie A. Wright, Tian Lin, Kathleen Krol, Allison M. Perkeybile, Hans P. Nazarloo, C. Sue Carter, Jessica Connelly, Natalie C. Ebner

**Affiliations:** 1 Department of Psychology, University of Florida, Gainesville, FL USA; 2 Department of Psychology, University of Virginia, Charlottesville, VA USA; 3 Kinsey Institute, Indiana University, Bloomington, IN USA; 4 Center for Cognitive Aging and Memory, McKnight Brain Institute, University of Florida, Gainesville, FL USA

**Keywords:** Plasma Oxytocin, Empathy, Oxytocin receptor gene, Methylation, Aging, Human behaviour, DNA methylation, Peptide hormones

## Abstract

Oxytocin (OT) levels in blood plasma and OT receptor gene (*OXTR*m) methylation are two crucial pieces of the endogenous OT system that have been independently associated with social cognition. Empathy is a social-cognitive skill essential for understanding others’ emotions and intentions, making it vital for everyday social interactions across the lifespan. Associations of plasma OT and *OXTR*m levels with empathy are currently not well understood among older adults, despite this period being marked by changes in both the endogenous OT system and empathy. Tying these previously separate lines of research together, this study examined the interplay of plasma OT and *OXTR*m on empathy in generally healthy older adults. Plasma OT levels were not directly related to empathy but for older adults with higher *OXTR*m levels higher plasma OT levels were associated with lower empathy scores. These results contribute to a growing literature on the intricate role of the endogenous OT system in social cognition in aging.

**Supplementary Information:**

The online version contains supplementary material available at 10.1038/s41598-025-07353-3.

## Introduction

Oxytocin (OT) is a neuropeptide that modulates social cognition^1,2^including empathy^3^which is the capacity to have an affective response to perceived, inferred, or imagined emotional states of others^4,5^. Several mechanisms have been proposed by which OT may modulate empathy, including facilitation of emotion perception^6^increasing feelings of secure social attachment^7^and stress regulation (e.g., promoting a sense of calm that allows individuals to focus on others rather than being preoccupied with their own stressors)^8^. Empathy is fundamental to maintaining satisfying social relationships^9^ but changes with age^10^ in that older adults are less able to understand the feelings of others^11^. Integrating research on empathy in aging with research on OT, here we examined for the first time the interplay between OT and OT receptor gene methylation (*OXTR*m), as two important features of the endogenous OT system, in their impact on empathy in older adults.

OT is produced primarily in magnocellular neurons of the hypothalamus^12^ and secreted both centrally throughout the brain and peripherally into the bloodstream. While the majority of OT is synthesized centrally, it is also synthesized in peripheral organs such as the heart, corpus luteum, adrenal glands, and dermis^13^. Centrally, OT acts on brain regions related to empathy, including the amygdala and other limbic regions^14^supporting its neuromodulatory function. Peripherally, OT acts on smooth muscles and regulates metabolism, labor, and cardiac activity^15^. Peripheral OT levels have been shown to trigger afferent branches of the vagus nerve that transmit signals to the brain prompting central OT release^16,17^which may constitute a physiological mechanism underlying OT’s role in social cognition, including empathy^18–20^.

Evidence from previous work on the relation between endogenous OT and empathy is mixed. In particular, greater plasma/salivary OT reactivity to emotional stimuli has been associated with greater empathy. For example, after watching an emotional video (i.e., a father describing his experience with his terminally ill child), plasma OT levels significantly increased in a sample of young adults and were associated with higher self-reported empathy^18^with comparable findings for salivary OT^19^. Extending this paradigm to a sample from a wide age range (18–99 years), this positive association between greater OT reactivity and higher empathy was most pronounced among older adults^20^. However, a recent systematic review of six studies found no link between endogenous OT and self-reported empathy^3^. Of note, however, the studies in this review varied largely in methodology, with four comprising healthy younger to middle-aged adults and two clinical samples (i.e., schizophrenia and craniopharyngioma); and four of them measured OT in plasma while two in saliva. Further, not considered in the review, the only study that included an adult lifespan sample (M age = 46.54 ± 16.30 years) found that lower salivary OT was associated with less self-reported empathy in individuals with hypopituitarism, but there was no association in healthy controls^21^.

Critically, most of the research has focused on peripheral OT levels, overlooking the OT receptor and its interplay with OT; which may be crucial for understanding individual differences in OT function, especially in aging. This is especially relevant as OT’s actions are largely dependent on engagement with receptors in both the central and peripheral nervous system^22,23^ which modulate physiological and psychological processes, including empathy^24^. The OT receptor, a G-protein coupled receptor with seven transmembrane domains, varies widely between individuals in part due to the expression of the gene located on chromosome 3p25.3 (*OXTR*)^22^. Epigenetic mechanisms, which are dynamic and reversible changes to the genetic DNA sequence due to environmental and socioemotional influences^25^can alter *OXTR*. Specifically, methylation of the cytosine-guanine dinucleotides in *OXTR* inhibits transcription and reduces OT receptor synthesis. Within *OXTR*, cytosine-phosphate-guanine (CpG) site − 934 has shown wide between-person variability, also in the context of aging^23,26^. Higher levels of *OXTR*m at this particular site have been linked to reduced sensitivity to endogenous OT as well as impaired ability to attend to socially relevant information^23,26–28^.

Given *OXTR*m’s role in OT receptor regulation and growing evidence of its impact on social-cognitive processes, *OXTR*m may modulate empathy. In fact, lower *OXTR*m levels were associated with greater self-reported empathy in healthy young adults^29^; and greater *OXTR*m levels in conduct disorder^30^ and psychopathy^31^ were associated with lower empathy. Less is known, however, about *OXTR*m in modulating empathy in aging, a period marked by substantial variability in *OXTR*m levels at CpG site − 934^23,26^. In fact, large late-life variance in *OXTR*m may constitute a biological mechanism underlying age-related changes in empathy and play a critical role in modulating OT’s effects on empathy in older adults.

Despite the literature discussing individual components of the endogenous OT system in the context of empathy, no study has examined both hormonal levels and receptors together. Specifically, as *OXTR*m increases, there are fewer receptors available to bind to the endogenous OT circulating in the body and the brain. Further understudied is the role of plasma OT and *OXTR*m, and their interplay, in empathy among older adults; a topic of relevance given that the ability to understand emotions and thoughts of others declines with age^9^. Furthermore, there is emerging evidence that older compared to younger age is associated with lower OT levels in blood serum^32–34^. Additionally, *OXTR*m may decline with age as evidence of decreased global DNA methylation in aged tissue suggests^35,36^.

To address these significant gaps in the literature, here we examined associations between plasma OT and empathy among generally healthy older adults; and determined the interplay between plasma OT and *OXTR*m in this context.

## Methods

### Participants

This study comprised 129 older adults (31% women, 92.3% White, aged *M* = 71.24 years, *SD* = 7.57 years, range = 55 to 94 years) from a larger clinical trial investigating the effects of a four-week intranasal OT administration on physical, cognitive, and socioemotional functioning in older adults (OT Aging Study; NCT0206943; for details of the larger clinical trial^37^). Participants with complete data for central variables under consideration here were included in the analysis. Recruitment took place between February 2016 and February 2020 throughout Alachua County and surrounding areas. Participants were recruited using community recruitment services at the University of Florida such as HealthStreet, via fliers and handouts in the community, mailouts to purchased addresses, phone calls to individuals in IRB-approved participant registries, advertisements on research websites (e.g., clinicaltrials.gov), the radio, and newspapers, as well as word of mouth. Upon study completion, participants received up to $350 compensation.

Eligibility criteria included: 55 years and older, generally healthy (as determined by a licensed clinician who reviewed self-reported health information during the initial in-person visit with the participant and which also included results from a Comprehensive Metabolic Panel and urine sampling), normal cognitive functioning (Telephone Interview of Cognitive Status^38^ scores *≥* 30), fluent in English, and able to provide informed written consent. Exclusion criteria included: hypersensitivity to OT or vasopressin, use of antidiuretic medications or a history of inappropriate hormone secretion, the combination of blood sodium levels < 134mEq/L with urine osmolality > 1200 L, a history of brain surgery or any serious brain damage or disease like aneurysm, stroke, or seizures, major medical surgery in the past two months, and heavy alcohol and/or drug use. See also Rung et al.^37^ for further details on inclusion/exclusion. Table 1 provides descriptive information for our analysis sample.

**Table 1:** Sample-descriptive information (*N* = 129): means, standard deviations, median, and range for demographics, health, mood, and cognition.

Measures	M (SD)	Median	Range
**Demographics**			
Age	71.24 (7.57)	70.55	55.87–94.81
Education	16.48 (3.45)	16	12–27
***Health***			
Mental	8.60(1.17)	9	5–10
Physical	8.16 (1.36)	8	4–10
***Mood***			
Positive Affect	3.47 (0.64)	3.54	1.54–4.85
Negative Affect	1.39 (0.39)	1.23	1.00–2.46
***Cognition***			
Crystallized	119.32 (11.82)	119	95.00–167.00
Fluid	89.75 (9.51)	90.13	63.00–116.03

*Notes.* Age: in years. Education: self-reported number of years of formal education. Health: Mental (*Please rate your general mental health*) and Physical (*Please rate your general physical health/mood*) on a scale from 1 = poor to 10 = excellent. Mood: Positive and Negative Affect Schedule (PANAS; 20-item short version^39^and 6 additional adjectives^40^), in general/on average, on a scale from 1 = very slightly or not at all to 5 = extremely. Cognition: NIH Cognition Toolbox^41^Crystallized and Fluid (uncorrected composite scores; normative mean = 100, standard deviation = 15). *M* = mean, *SD* = standard deviation.

Results of a sensitivity analysis using G*Power (version 3.1.9.7) for multiple linear regression with three predictors (i.e., plasma OT, *OXTR*m, and their interaction) supported our sample size of 129 participants with ɑ = 0.05 had a power of 80% to detect an effect of *f*^*2*^ = 0.088 (small to medium effect size)^42^.

## Study design

Rung et al. provides details and a flow chart of the larger clinical trial which comprised an initial phone screening, followed by an in-person screening visit, three pre-intervention visits, an intervention period of four-weeks during which participants self-administered intranasal oxytocin or a placebo, and three post-intervention visits^37^. For those who were eligible to undergo MRI, there was a fourth pre- and post-intervention visit (also see^43–46^ for published work from the larger trial). Data collection took place at the Department of Psychology, the Institute on Aging, and the McKnight Brain Institute at the University of Florida. Test sessions lasted ~ 2–3 h each. Participants were reimbursed for participation. The protocol was approved and monitored by the university’s Institutional Review Board, a Data Safety Monitoring Board, and the FDA. All procedures and methods were performed in accordance with guidelines and regulations.

Data analyzed in this paper were collected during the in-person screening visit and the third pre-intervention visit. Participants provided demographic and health information along with urine and blood samples during the in-person screening visit and met with a licensed clinician for a brief physical health exam to confirm study eligibility. The NIH Toolbox Cognition^41^ was also administered during this visit. The third pre-intervention visit occurred a few days later where participants completed several questionnaires, including the Empathy Quotient Short^47^ (EQ-Short).

## Measures

### Plasma OT

Intravenous blood was collected in 10 mL EDTA vials by a trained phlebotomist. Blood sampling was standardized to occur in the mornings (between 8:00am – 11:00am). Directly following blood draws, plasma was aliquoted by centrifuging the vials at 2300 rpm with a force of 1600×g at a temperature of 4 °C. Separated plasma was stored in a refrigerator at − 80 °C prior to assaying. Due to scheduling logistics, some samples (17.5%) were obtained between 11am – 1pm, and a few (6.2%) between 1pm – 3pm; plasma OT and OXTRm values did not differ across these sampling times (*p* > 0.05).

Enzyme immunoassay (EIA; Enzo Life Sciences, Inc.) was performed following all EIA kit instructions on unextracted samples. When OT is measured from blood plasma or saliva, extraction is a process whereby non-OT molecules are removed^48^. Several studies suggest that unextracted OT, which includes additional macromolecular species, shows a stronger relationship with behavior^20,49,50^ (for additional discussion regarding differences between extracted vs. unextracted plasma OT measurement, see^49,51^). As EIA is sensitive with a reported minimal detection rate of 15.6 micrograms per mL for OT and has minimal cross-reactivity for other neuropeptides, this assay is a reliable measure of plasma OT. To ensure reliability of the standard curve linear portion, all samples were first diluted with an assay buffer at a ratio of 1:8 (as directed by the immunoassay kit instructions) and all samples were run at the same time. This procedure produced coefficients of variance less than 10. Table 2 presents means, standard deviations, and ranges for plasma OT levels.

## Oxytocin receptor gene methylation

Intravenous blood was collected in 10 mL EDTA vials by a trained phlebotomist. Directly following blood draws, samples were centrifuged and approximately 1 mL of buffy coat was aliquoted and stored at − 80 °C until DNA extraction. DNA was isolated using Gentra Puregene Blood Kits (Qiagen, Hilden, Germany) with 100 nanograms (quantitated using Nanodrop) undergoing bisulfite conversion using MECOV50 Kits (Thermo Fisher Scientific, Waltham, USA). All following steps on this bisulfate-converted DNA were performed in triplicate.

Forty nanograms of bisulfite-converted DNA was subjected to amplification using polymerase chain reaction (PCR) using PyroMark PCR Kits (QIAGEN, Hilden, Germany) and 0.2 micromolar primers [TSL101F, 5′-TTGAGTTTTGGATTTAGATAATTAAGGATT-3′ (forward); TSL101R, 5′-biotin-AATAAAATACCTCCCACTCCTTATTCCTAA-3′ (reverse)]. Each PCR plate contained methylation standards (0, 50, and 100% methylated) and negative controls from bisulfite conversion and PCR. Thermocycling was performed as follows: Steps (i) 95 °C for 15 min; (ii) 50 cycles at 94 °C for 30 s, 56 °C for 30 s, and 72 °C for 30 s; (iii) 72 °C for 10 s; and (iv) 4 °C until analysis. A 116–base pair region (hg38 chr3: 8,769,044 − 8,769,159) was amplified on the coding strand of *OXTR* containing CpG-934 (hg38 chr3:8,769,121 − 8,769,122) and confirmed by agarose gel electrophoresis. DNA methylation level for each sample was assessed using pyrosequencing (PyroMark Q24, QIAGEN; sequencing primer: TSL101S, 5′-AGAAGT TATTTTATAATTTTT-3′). Mean deviation within replicates averaged ± 1.26%. Control samples generated appropriate methylation results (0% mean = 2.47% +/−0.12%; 100% mean = 99.62% +/−0.50; known mean = 44.22% +/−0.74). Table 2 presents means, standard deviations, and ranges for *OXTR*m levels.

### Empathy

Participants completed the EQ-Short^47^which comprises 22 items (selected from the original 40-item Empathy Quotient^52^; e.g., *Other people tell me I am good at understanding how they are feeling and what they are thinking; I can pick up quickly if someone says one thing but means another*) and has good internal validity as well as robust test-retest reliability^47^. Participants responded to each item on a scale from 1 (*strongly disagree*) to 5 (*strongly agree*), with higher scores indicating greater empathy. The total score for empathy was computed as the mean across all 22 items. Overall, the EQ showed good reliability in our sample of older adults with Cronbach’s α = 0.83 (*M* = 3.45, *SD* = 0.40). Table 2 presents means, standard deviations, and ranges for empathy scores.

**Table 2:** Descriptive statistics (mean (standard deviation), median, and range) for plasma OT, *OXTR*m, and empathy (*N* = 129).

Measures	M (SD)	Median	Range
**Predictors**			
Plasma OT	1542.36 (593.25)	1461	532–3288
*OXTR*m	49.73 (11.23)	50.11	15.13–80.76
***Outcome Variable***			
Empathy	3.50 (0.51)	3.55	2.05–4.73

*Notes.* Plasma Oxytocin (OT): in microgram per milliliter quantified in blood plasma via Enzyme Immunoassay (EIA; Enzo Life Sciences, Inc.). Oxytocin Receptor Gene Methylation (*OXTR*m): percent methylated (0–100), measured via pyrosequencing of DNA methylation from blood samples. Empathy: Empathy Quotient Short (EQ-Short), mean score across 22 items. *M* = mean, *SD* = standard deviation.

## Analysis

Univariate outliers were defined as values ± 3 standard deviations from the sample mean for plasma OT levels, *OXTR*m levels, and empathy scores. Following this criterion, data from two participants were removed from the analysis due to outlying values for plasma OT and one participant due to outlying values for *OXTR*m. Variables were z-standardized to aid interpretation of the results.

We used moderated regression analysis to test associations between plasma OT (continuous; range 532–3288 pg/mL) and empathy (continuous; range 1–5); and to determine the extent to which *OXTR*m (continuous; range 15.13–80.76%) moderated this association. In particular, plasma OT served as predictor, empathy as outcome, and *OXTR*m as moderator in this analysis. A significant main effect of plasma OT on empathy would support the association between plasma OT and empathy; and a significant plasma OT x *OXTR*m interaction on empathy the moderating role of *OXTR*m on the link between plasma OT and empathy.

Given the wide chronological age range in our sample (from 55 to 94 years), combined with growing evidence of age-differential effects of OT on social cognition^53^ as well as age-related differences in empathy^11^we controlled for age (continuous, in years) in our model. Given sex-dimorphic effects of OT on social cognition^54^we also controlled for sex (dichotomous; female vs. male; also see Table S2 in the Supplementary Materials for results from an exploratory model with age and sex as moderators). Further, we controlled for self-reported physical health (continuous; range 1–10) given its change with age^55^ and years of education (continuous; range 12–27, as proxy for socioeconomic status)^56^ given its associations with empathy^57^. We conducted analyses with and without participants who reported use of hormone replacement therapy (HRT; 2 participants; dummy coded)^58^ and selective serotonin reuptake inhibitors (SSRIs; 20 participants; dummy coded)^59^ given their possible interactions with the OT system. Results from analyses with and without covariates included/cases removed were consistent (see Table S1 in the Supplementary Materials for results for models with/without control variables). In-text, we report results from the model that controlled for age, sex, physical health, education, HRT use, and SSRI use. All data and analysis code are archived under https://osf.io/6nt2s/ (Polk et al., 2025).

## Results

**Fig. 1:**
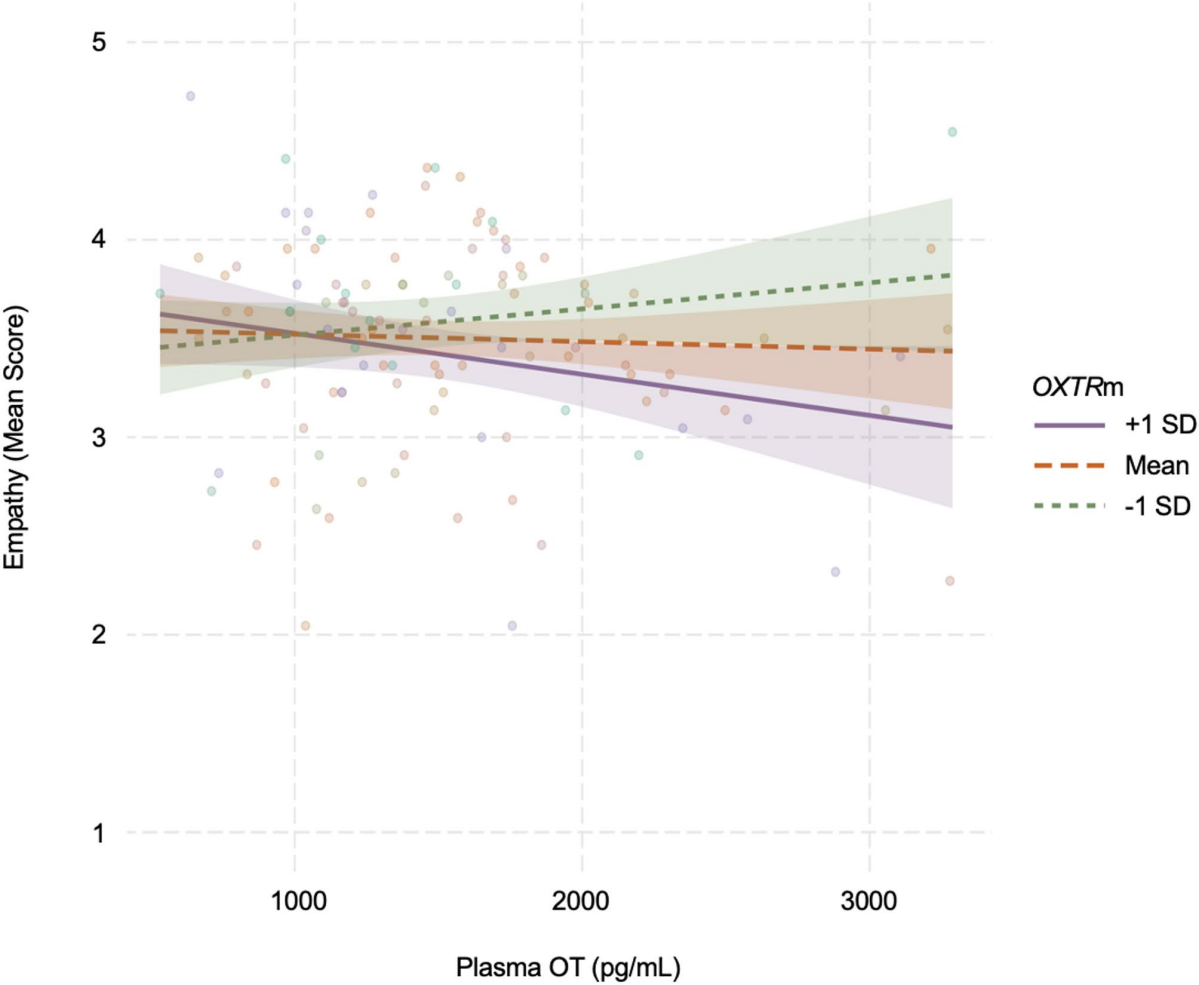
The association between plasma OT levels and empathy scores was moderated by *OXTR*m levels. In particular, the relationship between plasma OT and empathy was negative as *OXTR*m increased.

*Notes.* OT = Oxytocin; *OXTR*m = Oxytocin Receptor Gene Methylation; M = Mean; SD = Standard Deviation. Dots represent individual data points. Regression fit lines are shown at the mean and ± 1 standard deviation of the moderator (*OXTR*m), with colored bands (green = low *OXTR*m, orange = mean *OXTR*m, purple = high *OXTR*m) representing 95% Confidence Intervals.

The moderated linear regression analysis to examine associations of plasma OT on empathy and the role of *OXTR*m on this association, controlling for age, sex, physical health, education, HRT use, and SSRI use, was significant (*F*(9, 108) = 3.413, *p* < 0.001, adjusted R² = 0.157). The main effect of plasma OT on empathy was not significant (*B* = −0.022, *SE* = 0.048, *t*(108) = −0.468, *p* = 0.640). *OXTR*m was trendwise negatively associated with empathy (*B* = −0.084, *SE* = 0.044, *t*(108) = −1.922, *p* = 0.057), qualified by a significant interaction between plasma OT and *OXTR*m on empathy (*B* = −0.097, *SE* = 0.041, *t*(108) = −2.357, *p* = 0.020). In particular, as shown in Fig. 1, when *OXTR*m levels were high, the relationship between plasma OT and empathy was negative, supporting *OXTR*m levels as moderator in the association between plasma OT and empathy in our sample of older adults.

There was a significant effect of sex on empathy (*B* = 0.136, SE = 0.050, *t*(108) = 0.136, *p* = 0.008), in that women compared to men reported higher levels of empathy None of the other control variables were significant (age: *B* = −0.080, *SE* = 0.051, *t*(108) = −1.569, *p* = 0.120; physical health: *B* = 0.086, *SE* = 0.047, *t*(108) = 1.851, *p* = 0.669, education: *B* = 0.036, *SE* = 0.049, *t*(108) = 0.729, *p* = 0.468, HRT use: *B* = 0.039, *SE* = 0.044, *t*(108) = 0.870, *p* = 0.386, SSRI use: *B* = −0.031, SE = 0.048, t(108) = −0.638, *p* = 0.525). See Table S1 in the Supplementary Materials for results for models with/without control variables, which converge with results reported in-text. Further, while the current study was not sufficiently powered to systematically investigate moderation by age and/or sex, Table S2 in the Supplementary Materials presents results from an exploratory analysis into age/sex moderations.

## Discussion

This study examined the relation between plasma OT and empathy in generally healthy older adults, and for the first time, determined the interplay between plasma OT and *OXTRm*, as two crucial components of the endogenous OT system, on empathy. While we did not observe a direct relationship between plasma OT and empathy in our sample of older adults, we found that *OXTR*m moderated the association between plasma OT and empathy: for higher *OXTR*m levels the association between plasma OT and empathy was negative. These key results of the study are discussed next.

The finding that plasma OT was not associated with empathy in our older adults aligns with several studies in young adults that suggest that peripheral endogenous OT and self-reported empathy were not associated^3,60^. Thus, our findings contribute to a growing literature supporting that plasma/salivary OT levels may not be directly associated with empathy in healthy individuals when using self-reported measures. Our findings, however, differ from initial evidence on plasma/salivary OT reactivity to empathy-inducing stimuli in both young^18,19^ and older^20^ adults showing that greater OT release was associated with greater empathy.

The lack of a direct relationship between plasma OT and empathy in the present study and consistent previous work^3,60^ suggests that basal OT levels may not be related to trait empathy – a general trait, not context-dependent, and rather stable over time^61^; and which is commonly measured using self-report scales including the EQ-Short^47^ as in the present study. In contrast, OT reactivity to state empathy as assessed in studies that lead to results divergent from ours^18–20^conceptualize empathy as dynamic in response to situational factors^61^. Though speculative at this point, differential activation of the OT system in response to situational stimuli (e.g., emotional videos)^18–20^ vs. stable, trait-like expression (i.e., trait empathy)^3,60^ may underlie differential results pattern across studies. That is, basal OT levels, at rest and not in response to specific stimulation, may be different from stimulus-induced, context-dependent fluctuations in the OT system that drive empathy. OT reactivity may be a more sensitive measure in the study of state-like empathetic response and should be explored further in aging.

Some literature differentiates between two dimensions of empathy: cognitive empathy which refers to the ability to understand others’ emotions vs. affective empathy which refers to one’s own emotional experience in response to an emotional stimulus^61^; with cognitive (but not affective) empathy demonstrating age-related decline^11^. Even more, differential associations between plasma OT levels and these two facets of empathy have been suggested, with greater plasma OT reactivity associated with reduced cognitive^62^ but greater affective^18,63^ empathy. The EQ-Short used here, however, conceptualizes empathy unidimensionally, and it is possible that our operationalization lacked the sensitivity to capture the complex role plasma OT plays in different facets of empathy among older adults.

Importantly, and advancing understanding of the mechanisms by which OT modulates empathy, we did observe that *OXTR*m moderated the association between plasma OT and empathy. This observation aligns with literature showing that clinical conditions such as psychopathy^31^ and conduct disorders^30^ are marked by both increased *OXTR*m levels and decreased empathy. While the present study excluded for serious brain damage or disease like aneurysm, stroke, or seizures as well as heavy alcohol and/or drug use, we did not specifically exclude individuals with neurological and psychiatric diseases; and thus cannot speak to variations between individuals with and without these conditions, given their possible impact on endogenous OT system and function^64^. Future research would benefit from consideration of mental health status to delineate the interplay between plasma OT, *OXTR*m, and empathy among these populations in aging.

Our results extend previous findings to older adults in support of a role of *OXTR*m in the link between plasma OT and empathy. In fact, higher *OXTR*m levels may activate a negative feedback loop, resulting in a negative association between plasma OT and empathy, as observed in our study. More specifically, higher *OXTR*m levels reduce *OXTR* expression, limiting the availability of OT receptors to bind^25,65^. In turn, elevated *OXTR*m levels may disrupt OT’s function in social-cognitive processes, like empathy, which could explain the negative association between plasma OT and empathy we observed here. That is, when *OXTR*m levels are high, OT’s capacity to enhance empathy may be impaired, despite high plasma OT levels. This process may be exacerbated in older adults as binding between OT and its receptor is diminished in advanced age^66^. Future research will be able to systematically test this possible explanation.

The present study’s extension of research into older adults is crucial, given that associations of the endogenous OT system with empathy has so far almost exclusively been conducted in healthy young adults or in young to middle-aged clinical samples; despite evidence of plasma OT changes^32–34^ and changes in OXTRm^23,26,67^ with age. Our findings help to fill a significant gap in the literature by providing first insights into how the OT system influences empathetic processes in older adults, speaking to the biological basis of empathy in aging by integrating hormonal and epigenetic mechanisms. This investigation will benefit from incorporating longitudinal designs on the interplay between plasma OT, *OXTR*m, and empathy across the adult age range. These novel research directions will advance biological theories of social cognition in aging.

In fact, it is possible that the interplay between plasma OT and *OXTR*m on empathy becomes particularly relevant with advanced age. Supporting this, here we observed a large degree of variability in *OXTR*m within our sample of generally healthy older adults (range = 15–81%), in line with two other studies that also found a large *OXTR*m variability at CpG site − 934 in older adults and also found that older adults were significantly more variable in *OXTR*m than younger adults^23,26^. These ranges are also larger than those previously reported in children (36 − 54% in children aged 5–11 years)^68^infants (41 − 74%, aged 18 months)^69^ or mothers of infants (51–81%)^70^. This large variability in *OXTR*m at site − 934 in older adults could be a result of heterogeneous experiences over the course of life^23^. More specifically, epigenetic regulation of *OXTR* via changes in *OXTR*m facilitates flexibility and adaptation of the OT system to the environment, particularly during early childhood, reflecting biological modulation of social processes^71^. In young adulthood, *OXTR*m levels stabilize^23,72^becoming less dynamic in their responses to environmental changes. Given the substantial variability in *OXTR*m at site − 934 observed in older adults, however, it is possible that continued environmental experiences contribute to shaping *OXTR*m patterns as time progresses across the lifespan with lasting effects on social behavior and social cognition, including empathy. In this context, our results suggest that the interplay between *OXTR*m and plasma OT on empathy in older age may reflect accumulated environmental influences over time, a proposition that future research will be able to confirm in longitudinal studies.

### Limitations

Due to logistics of the larger clinical trial^37^study blood samples for determination of plasma OT and *OXTR*m levels were collected on a different day than the empathy data. Although plasma OT levels have been shown to be relatively stable across days^73,74^there is work that fluctuations in plasma OT levels occur as a function of hydration levels, physical activity, and sleep^67^. Furthermore, repeated sampling of both endogenous OT markers and empathy would have allowed for a more robust capture of our central constructs of interest for a finer characterization of their interplay and over time. Also, as measurement of central OT is invasive requiring lumbar puncture, here we measured peripheral OT levels in blood plasma. While some research supports a high correlation between central and peripheral OT levels (*r* = 0.80)^75^, future work sampling OT in cerebrospinal fluid would directly inform central OT mechanisms in their interactions with *OXTR*m and impact on empathy among older adults. Additionally, there is evidence of age and sex effects on empathy^10,11^ as well as on plasma OT^32–34^ and OXTRm^23,26^. The sample size of the present study did not allow for a systematic test of age and/or sex moderations of our findings (but see results of exploratory analyses of age and sex moderations in Table S2 of the Supplementary Materials). A well-powered systematic analysis of age and sex, and their interplay, on the associations between plasma OT, *OXTR*m, and empathy in older adults is warranted in future extensions of this research.

## Conclusions

While plasma OT was not directly related to empathy in our sample of generally healthy older adults, it interacted with *OXTR*m in their impact on empathy. Specifically, with higher levels of *OXTR*m, the relation between plasma OT and empathy was negative. Reduced receptor availability, due to greater *OXTR*m levels, may disrupt signaling within the OT system, activating a negative feedback loop. Integrating disparate lines of research for the first time, findings from our work provide novel insight into the biological basis of empathy, suggesting the endogenous OT system as a crucial factor in social-cognitive aging. Broadly, our findings provide novel evidence underscoring the importance of integrating hormonal and epigenetic measures in social-cognitive aging research. Results reported here further understanding of the biological mechanisms underlying social behaviors and affiliative processes in later life and can inform future interventions aimed at enhancing empathy and strengthening social bonds in aging.

## Supplementary Information

Below is the link to the electronic supplementary material.

Supplementary material 1

## Data Availability

The datasets and code for analysis used in the present study are available in the open science framework repository, https://osf.io/6nt2s/.
