# Supplementary material for: Interplay of plasma Oxytocin and oxytocin receptor gene methylation levels on empathy in older adults
Source: Sci Rep. 2025 Jul 18;15:26057. doi: 10.1038/s41598-025-07353-3 (PMC12274532; doi:10.1038/s41598-025-07353-3)
Supplement: Supplementary file 1 — Supplementary material 1 [file 41598_2025_7353_MOESM1_ESM.pdf]

Supplementary Materials

Table S1. Results for moderated linear regression models with/without control variables (*in italics*).

**Predictors**	**Model 1** (n = 129)	**Model 2** (n = 129)	**Model 3** (n = 118)	**Model 4** (n = 96)
Intercept	**3.50 (0.044)**	**3.50 (0.043)**	**3.516 (0.045)**	**3.522 (0.049)**
Plasma OT	-0.066 (0.044)	-0.044 (0.045)	-0.022 (0.048)	-0.048 (0.052)
*OXTR*m	-0.081 (0.044)	-0.085 (0.043)	-0.084 (0.044)	**-0.105 (0.048)**
Plasma OT x *OXTR*m	**-0.128 (0.041)**	**-0.122 (0.040)**	**-0.097 (0.041)**	**-0.114 (0.043)**
Age	--	*-0.025 (0.044)*	*-0.081 (0.051)*	*-0.062 (0.055)*
Sex	--	***0.105 (0.045)***	***0.134 (0.050)***	***0.145 (0.054)***
Physical Health	--	--	*0.086 (0.047)*	*0.088 (0.050)*
Education	--	--	*0.036 (0.049)*	*0.042 (0.054)*
HRT	--	--	*0.038 (0.044)*	--
SSRI	--	--	*-0.031 (0.048)*	--
**Adjusted R^2**	**0.081**	**0.113**	**0.161**	**0.185**

NOTE: Unstandardized B coefficients and standard errors (*B*(*SE*)) are reported. Education (in years). OT = oxytocin, HRT = hormone replacement therapy, SSRI = selective serotonin reuptake inhibitor. Bold indicates $p < 0.050$.

In these moderated linear regression models plasma OT served as predictor, *OXTR*m as moderator, and empathy as outcome. Age, sex, physical health, education, HRT use, and SSRI use served as covariates. In all models, outliers were identified as observations exceeding ±3 standard deviations from the mean and were excluded from analyses; this approach resulted in the removal of two participants due to outlying plasma OT values and one participant due to an outlying *OXTR*m value.

Model 1 did not contain any control variables.

Model 2 controlled for age (continuous; in years) and sex (dichotomous; female vs. male), given our sample's wide chronological age range (55-94 years), evidence of age-related differences in empathy[1] and of age-differential[2] as well as sex-dimorphic[3] social-cognitive effects of OT.

Model 3 was identical to Model 2 but also controlled for physical health (continuous; 1-10), education (continuous; 12-27 years; as proxy for socioeconomic status)[4], HRT use (dummy coded), and SSRI use (dummy coded). Physical health typically decreases with age[5];

socioeconomic status has been negatively associated with empathy[6]; HRT and SSRI use have been shown to interact with the OT system[7,8]. Eleven participants did not have data for education.

Model 4 was identical to Model 3 but removed two participants who reported HRT use and 20 who reported SSRI use. As in Model 3, an additional 11 participants did not have data for education.

Effects of interest were comparable across the four models. Model 3 results are reported in-text.

Table S2. Result for moderated linear regression model with sex and age as additional moderators (*in italics*).

**Predictors**	**Model**<br>(n = 118)
Intercept	**3.528 (0.055)**
Plasma OT	-0.020 (0.059)
*OXTR*m	-0.088 (0.063)
Plasma OT x *OXTR*m	**-0.166 (0.073)**
Age	-0.087 (0.057)
Sex	0.119 (0.064)
Physical Health	**0.105 (0.050)**
Education	0.050 (0.058)
HRT	0.052 (0.046)
SSRI	-0.010 (0.053)
Age x Sex	*-0.026 (0.062)*
Plasma OT x Age	*0.007 (0.055)*
OXTRm x Age	*0.021 (0.117)*
Plasma OT x OXTRm x Age	*0.121 (0.118)*
Plasma OT x Sex	*0.049 (0.073)*
OXTRm x Sex	*-0.029 (0.077)*
Plasma OT x OXTRm x Sex	*-0.111 (0.093)*
Plasma OT x Age x Sex	*-0.061 (0.062)*
OXTRm x Age x Sex	*-0.118 (0.160)*
Plasma OT x OXTRm x Age x Sex	*0.220 (0.167)*
**Adjusted R^2**	**0.152**

NOTE: Unstandardized B coefficients and standard errors (*B*(*SE*)) are reported. Education (in years). OT = oxytocin, HRT = hormone replacement therapy, SSRI = selective serotonin reuptake inhibitor. Bold indicates $p < 0.050$.

In exploratory fashion, we added chronological age and sex as additional moderators to the moderated linear regression model described above with physical health, education, HRT use, and SSRI use serving as covariates.

Effects of interest were unchanged with age, sex, and their interaction added as additional moderators; and none of the moderator effects were significant.

References:

1 Beadle, J. N. & de la Vega, C. E. Impact of Aging on Empathy: Review of Psychological and Neural Mechanisms. *Frontiers in Psychiatry* **10** (2019). https://doi.org:10.3389/fpsyt.2019.00331

2 Horta, M., Pehlivanoglu, D. & Ebner, N. C. The Role of Intranasal Oxytocin on Social Cognition: An Integrative Human Lifespan Approach. *Curr Behav Neurosci Rep* **7**, 175-192 (2020). https://doi.org:10.1007/s40473-020-00214-5

3 Lu, Q. *et al.* Sexual dimorphism of oxytocin and vasopressin in social cognition and behavior. *Psychol Res Behav Manag* **12**, 337-349 (2019). https://doi.org:10.2147/prbm.S192951

4 Sirin, S. R. The relationship between socioeconomic status and school outcomes [microform]: Meta analytic review of research. *Rev Educ Res* **75**, 417-453 (2005).

5 Luo, M. S. & Li, L. W. Are Self-perceptions of Aging Associated With Health Trajectories Among Middle-Aged and Older Adults? *The Gerontologist* **60**, 841-850 (2019). https://doi.org:10.1093/geront/gnz092

6 Sommerlad, A., Huntley, J., Livingston, G., Rankin, K. P. & Fancourt, D. Empathy and its associations with age and sociodemographic characteristics in a large UK population sample. *PLOS ONE* **16**, e0257557 (2021). https://doi.org:10.1371/journal.pone.0257557

7 Marazziti, D. *et al.* A link between oxytocin and serotonin in humans: Supporting evidence from peripheral markers. *European Neuropsychopharmacology* **22**, 578-583 (2012). https://doi.org:https://doi.org/10.1016/j.euroneuro.2011.12.010

8 Procyshyn, T. L., Watson, N. V. & Crespi, B. J. Experimental empathy induction promotes oxytocin increases and testosterone decreases. *Horm Behav* **117**, 104607 (2020). https://doi.org:10.1016/j.yhbeh.2019.104607
